# Biocompatibility effects of biologically synthesized graphene in primary mouse embryonic fibroblast cells

**DOI:** 10.1186/1556-276X-8-393

**Published:** 2013-09-23

**Authors:** Sangiliyandi Gurunathan, Jae Woong Han, Vasuki Eppakayala, Ahmed Abdal Dayem, Deug-Nam Kwon, Jin-Hoi Kim

**Affiliations:** 1 Department of Animal Biotechnology, Konkuk University, 1 Hwayang-Dong, Gwangin-gu, Seoul 143-701, South Korea

**Keywords:** Atomic force microscopy, Biocompatibility, Graphene oxide, Graphene, Scanning electron microscopy, UV-visible spectroscopy

## Abstract

Due to unique properties and unlimited possible applications, graphene has attracted abundant interest in the areas of nanobiotechnology. Recently, much work has focused on the synthesis and properties of graphene. Here we show that a successful reduction of graphene oxide (GO) using spinach leaf extract (SLE) as a simultaneous reducing and stabilizing agent. The as-prepared SLE-reduced graphene oxide (S-rGO) was characterized by ultraviolet–visible spectroscopy and Fourier transform infrared spectroscopy. Dynamic light scattering technique was used to determine the average size of GO and S-rGO. Scanning electron microscopy and atomic force microscopy images provide clear surface morphological evidence for the formation of graphene. The resulting S-rGO has a mostly single-layer structure, is stable, and has significant water solubility. In addition, the biocompatibility of graphene was investigated using cell viability, leakage of lactate dehydrogenase and alkaline phosphatase activity in primary mouse embryonic fibroblast (PMEFs) cells. The results suggest that the biologically synthesized graphene has significant biocompatibility with PMEF cells, even at a higher concentration of 100 μg/mL. This method uses a ‘green’, natural reductant and is free of additional stabilizing reagents; therefore, it is an environmentally friendly, simple, and cost-effective method for the fabrication of soluble graphene. This study could open up a promising view for substitution of hydrazine by a safe, biocompatible, and powerful reduction for the efficient deoxygenation of GO, especially in large-scale production and potential biomedical applications.

## Background

Recently, carbon-based nanomaterials such as carbon nanotubes, graphene oxide, and graphene have been explored extensively by researchers as well as the industry. Graphene is an emerging nanomaterial which has greater scientific and commercial advantages. Recently, single-layer and few-layer graphenes received great interest due to its exceptional characteristics including high surface area as well as strong electronic, mechanical, thermal, and chemical properties in various fields such as materials science, physics, chemistry, biotechnology, and nanomedicine [1-3]. Particularly, graphene has been attracted to the scientific community for numerous potential applications in biotechnology, including biosensing [4,5], disease diagnostics [6], antibacterial [7-11] and antiviral materials [12], cancer-targeting [13] and photothermal therapy [14,15], drug delivery [16-18], and tissue engineering [19,20] due to these unique physical, chemical, and biocompatibility properties. During the past few years, several procedures have been established for the synthesis of graphene and its derivatives, including mechanical exfoliation, epitaxial growth, unzipping carbon nanotubes, exfoliation of GO, and liquid-phase exfoliation of graphite [21]. Moreover, several other methods were implemented to prepare high-quality graphene such as chemical vapor deposition onto thin films of metal, epitaxial growth on electrically insulating surfaces like silicon carbide, and the scotch tape method [21]. All of these methods can produce highly crystalline graphene but are not suitable for mass production [22,23]. Several researchers have attempted to propose environmentally friendly and green approach including flash photo reduction [24] hydrothermal dehydration [22], solvothermal reduction [23], and catalytic [25] and photocatalytic reduction [26]. The most promising method for the large-scale production of graphene is the chemical oxidation of graphite, conversion of the resulting graphite oxide to GO, and subsequent reduction of GO. The exfoliation of GO is one of the well-established methods for the mass production of graphene in the presence of some chemical reducing agents such as hydrazine and sodium borohydride [27,28]. The usage of strong chemical reducing agents such as hydrazine is found to be corrosive, highly explosive, and highly toxic [29]. In addition, hydrazine seems to be a hepatotoxic and carcinogenic agent in the kidney, and liver damage can result in blood abnormalities, irreversible deterioration of the nervous system, and even DNA damage [30]. In this context, many studies used the green chemistry approach for the reduction of GO to overcome the toxicity problem using various biological molecules as reducing agents such as vitamin C [31], melatonin [32], sugars [33], polyphenols of green tea [34,35], bovine serum albumin [36], and biomass of bacteria [37,38]. The biologically derived graphene nanomaterials are biocompatible, stable, and soluble.

Biocompatibility is an essential factor for tissue engineering applications. Recent studies suggest that the biocompatibility of carbon-based nanomaterials depends strongly on mass, purity, ratio, and surface functional groups. A variety of biological applications depend on the functionalization of graphene. The ability of the functionalization of graphene and its derivatives brought the attention of nanomaterials in various applications including biosensors and tissue engineering. Several studies have reported the biocompatibility of graphene derivatives in proximity of mammalian cells. Biris et al. [39] demonstrated that osteoblast cells (MC3T3-E1) have a high ability to grow on graphene film. Agarwal et al. [19] reported that reduced graphene oxide (rGO) is more biocompatible than single-wall carbon nanotubes using different cell lines including neuroendocrine PC12 cells, oligodendroglia, or osteoblasts. Recently, Gurunathan and coworkers reported that microbially reduced graphene oxide shows significant biocompatibility with primary mouse embryonic fibroblast (PMEF) cells. Chen et al. [40] cultured PC12 cells with carbon nanotubes and rGO films with the same initial seeding density for 5 days, and the results suggest that the cells cultured with rGO enhanced proliferation, whereas nanotubes inhibited the proliferation of cells. Nayak et al. [41] reported that G-coated substrates accelerated osteogenic differentiation of human mesenchymal stem cells (hMSCs) compared to uncoated substrates. Lee et al. [42] reported that GO films enhanced adipogenesis of hMSCs due to their high affinity with insulin. Chen et al. [43] reported that G-coated substrates maintained induced pluripotent stem cells in the undifferentiated state.

Regarding synthesis of nanomaterials, various phytochemicals have been used from different natural sources like leaves, peels, roots, seeds, and other parts of plants as reducing agents for the synthesis of different metal nanoparticles like silver and gold [44-47]. Here we attempted to use spinach leaves because it is nontoxic and an edible plant which has high nutritional value and is extremely rich in antioxidants; therefore, the leaf extracts of spinach could be potential alternative reducing agents for the synthesis of soluble graphene. In the present report, we investigated a greener approach for the reduction of GO using spinach leaf extracts (SLE), and also we analyzed the biocompatibility effect of SLE-reduced graphene oxide (S-rGO) in PMEFs.

## Methods

### Chemicals

Graphite powder was purchased from Sigma-Aldrich (St. Louis, MO, USA). NaOH, KMnO_4_, anhydrous ethanol, 98% H_2_SO_4_, 36% HCl, and 30% hydrogen peroxide aqueous solution were purchased from Sigma-Aldrich (USA) and used directly without further purification. All aqueous solutions were prepared with deionized (DI) water. All other chemicals were purchased from Sigma-Aldrich (St Louis, MO, USA) unless stated otherwise.

Spinach leaves were obtained from the local market and stored at 4°C until needed. Twenty grams of spinach leaves was washed thoroughly with double distilled water and was then sliced with a sharp stainless steel knife into fine pieces, about 1 to 5 cm^2^. The finely cut spinach leaves were mixed in 100 mL of sterile distilled water and then boiled for 2 min. After boiling, it was filtered through Whatman filter paper no. 1. Further, the extracts were used for synthesis of graphene. The extracts were stored at 4°C until further use.

### Synthesis of GO

Natural graphite powder was utilized as the raw material to prepare graphite oxide by suspension through a modified Hummers’ method [48] and according to Esfandiar et al. [32]. The prepared graphite oxide powder was dispersed in DI water to obtain an aqueous graphite oxide suspension with a yellow-brownish color. The suspension was centrifuged at 3,000 rpm/min for 10 min to eliminate unexfoliated graphitic plates and then at 10,000 rpm/min for 10 min to remove tiny graphite particles. Finally, a GO suspension was achieved by exfoliation of the filtered graphite oxide suspension through its sonication. Reduction of graphene oxide was followed as described earlier [38] with slight modification.

### Synthesis of reduced graphene oxide

Reduced graphene oxide was obtained from the reaction of a plant extract with graphene oxide. In the typical reduction experiment, 10 mL of spinach leaf extract was added to 40 mL of 0.5 mg/mL aqueous GO solution and then the mixture was kept in a tightly sealed glass bottle and stirred at 30°C for 24 h. Then, using a magneto-stirrer heater, reduced graphene oxide suspension was stirred at 400 rpm at a temperature of 30°C for 30 min. A homogeneous S-rGO suspension was obtained without aggregation. Then, the functionalized S-rGO was filtered and washed with DI water. Finally, a black S-rGO dispersion was obtained.

### Characterization

Ultraviolet–visible (UV–vis) spectra were obtained using a WPA (Biowave II, Biochrom Cambridge, UK). The aqueous suspension of GO and S-rGO was used as UV–vis samples, and deionized water was used as the reference. The particle size of dispersions was measured by Zetasizer Nano ZS90 (Malvern Instruments Limited, Malvern, UK). X-ray diffraction (XRD) analyses were carried out on an X-ray diffractometer (Bruker D8 DISCOVER, Bruker AXS GmBH, Karlsruhe, Germany). The high-resolution XRD patterns were measured at 3 kW with Cu target using a scintillation counter, and *λ* = 1*.*5406 A at 40 kV and 40 mA was recorded in the range of 2*θ* = 5° − 80°. The changes in the surface chemical bonding and surface composition were characterized using a Fourier transform infrared spectroscopy (FTIR) instrument (PerkinElmer Spectroscopy GX, Branford, CT, USA). A JSM-6700F semi-in-lens FE-SEM operating at 10 kV was used to acquire SEM images. The solid samples were transferred to a carbon tape held by an SEM sample holder for analyses. The analyses of the samples were carried out at an average working distance of 6 mm. Raman spectra of graphene oxide and reduced graphene oxide were measured by WITec Alpha300 (Ulm, Germany) with a 532-nm laser. The calibration was initially made using an internal silicon reference at 500 cm^−1^ and gave a peak position resolution of less than 1 cm^−1^. The spectra were measured from 500 to 4,500 cm^−1^. All samples were deposited on glass slides in powder form without using any solvent. Surface images were measured using tapping-mode atomic force microscopy (SPA 400, SEIKO Instruments, Chiba, Japan) operating at room temperature. Height and phase images were recorded simultaneously using nanoprobe cantilevers (SI-DF20, SEIKO Instruments).

### Exposing PMEFs to GO and S-rGO

PMEF cells were routinely cultured in Dulbecco’s modified Eagle’s low-glucose medium (DMEM/low, Gibco BRL Life Technologies, Grand Island, NY, USA) supplemented with 10% (*v*/*v*) fetal bovine serum, plus 2 mM of l-glutamine, and 1% (*v*/*v*) penicillin-streptomycin (10 U mL^−1^ penicillin and 0.1 mg mL^−1^ streptomycin) and grown at 37°C in a 5% CO_2_ humidified environment. When the cells had reached 70% confluence, they were trypsinized (0.25% trypsin and 0.04% EDTA, Sigma-Aldrich) and passaged (1:3). Cells within three passages were used for experiments. GO or S-rGO suspensions were freshly prepared before the cells were exposed and diluted to appropriate concentrations from 20 to 100 μg mL^−1^ with the culture medium; they were then immediately applied to the cells. DMEM without GO and S-rGO supplements served as a negative control in each experiment.

### Cell viability assay

WST-8 assay was followed as described earlier by Liao et al. [49]. Typically, 1 × 10^4^ cells were seeded in a 96-well plate and cultured in DMEM supplemented with 10% at 37°C under 5% CO_2_. After 24 h, the cells were washed with 100 μL of serum-free DMEM two times and incubated with 100 μL of different concentrations of GO or S-rGO suspensions in serum-free DMEM. After a 24-h exposure, the cells were washed twice with serum-free DMEM, and 15 μL of WST-8 solution was added to each well containing 100 μL of serum-free DMEM. After 1 h of incubation at 37°C under 5% CO_2_, 80 μL of the mixture was transferred to another 96-well plate because residual GO or S-rGO can affect the absorbance values at 450 nm. The absorbance of the mixture solutions was measured at 450 nm using a microplate reader. Cell-free control experiments were performed to see if GO and rGO react directly with WST-8 reagents. Typically, 100 μL of GO or S-rGO suspensions with different concentrations (20 to 100 μg/mL) was added to a 96-well plate and 10 μL of WST-8 reagent solution was added to each well; the mixture solution was incubated at 37°C under 5% CO_2_ for 1 h. After incubation, GO or S-rGO was centrifuged and 50 μL of the supernatant was transferred to another 96-well plate. The optical density was measured at 450 nm.

### LDH assay

Cell membrane integrity of PMEF cells was evaluated by determining the activity of lactate dehydrogenase (LDH) leaking out of the cell according to manufacturer’s instructions (*in vitro* toxicology assay kit, TOX7, Sigma-Aldrich). The LDH assay is based on the release of the cytosolic enzyme, LDH, from cells with damaged cellular membranes. Thus, in cell culture, the course of GO- and S-rGO-induced cytotoxicity was followed quantitatively by measuring the activity of LDH in the supernatant. Briefly, cells were exposed to various concentrations of GO and S-rGO for 24 h, and then 100 μL per well of each cell-free supernatant was transferred in triplicates into wells in a 96-well plate, and 100 μL of LDH assay reaction mixture was added to each well. After 3 h of incubation under standard conditions, the optical density of the color generated was determined at a wavelength of 490 nm using a microplate reader.

### Alkaline phosphatase activity

PMEF cells were cultured in a 48-well culture dish at a density of 5 × 10^3^ cells per well for 4 days. Then medium was replaced with treatment solution, which was DMEM containing 5% serum plus GO or S-rGO. After 4 days, the alkaline-phosphatase activity was measured according to the method described by manufacturer’s instructions (DALP-250, QuantiChrom™ Alkaline Phosphatase Assay Kit, Gentaur, Belgium). The plates were incubated at 37°C for 30 min. The amount of released p-nitrophenol was measured at 405 nm in a 96-well microplate reader. Enzyme activity was evaluated as the amount of nitrophenol released through the enzymatic reaction, and absorbance was recorded using a microplate reader (Bio-RAD 680, Hercules, CA, USA) at 405 nm. For normalization, the total protein content was measured using a bicinchoninic acid protein assay kit. Thus, the alkaline phosphatase (ALP) activity was expressed and normalized by the total protein content (U/mg).

## Results and discussion

### Reduction of GO by SLE

**Figure 1:**
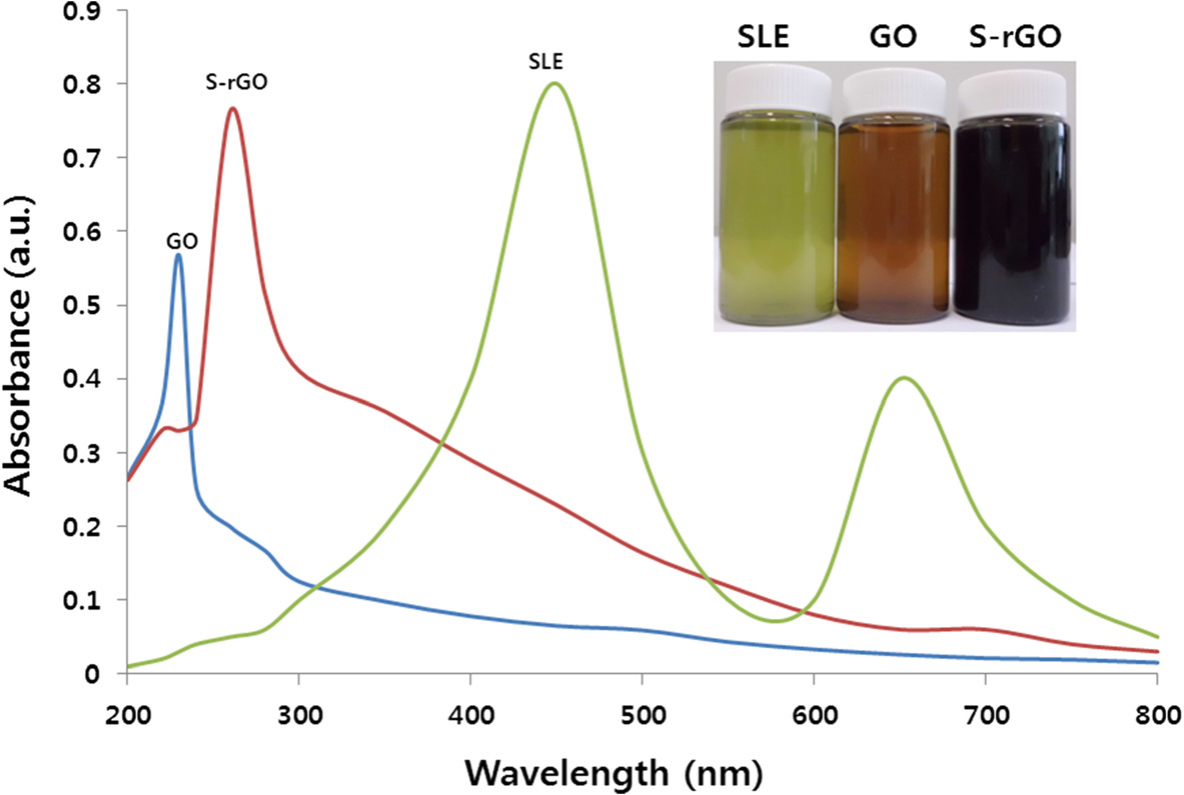
UV–vis absorption spectra of SLE, GO, and S-rGO suspensions in water.

Reduction of GO was carried out at room temperature using spinach leaf extract. On completion of the reduction process, the color change from brown to black provides the soluble reduced product (inset of the Figure 1). This preliminary experiment suggested that spinach leaf extracts have the ability to remove oxygen-containing moieties present in GO, which is the piece of evidence for reduction process. Further, the spectra of GO and S-rGO were recorded using UV–vis absorption spectroscopy, which is a simple and valuable technique. GO shows a maximum absorption peak at 231 nm which was attributed to the *π*-π* transitions of the aromatic C-C bonds and a weak shoulder at 300 nm due to *n*-π* transitions of C=O bonds. After complete reduction, a red shift of this characteristic peak was observed at 265 nm for S-rGO (Figure 1); this indicates that electronic conjugation was restored. When SLE was used as control, it showed two peaks at 450 and 650 nm, which are different from those of GO and S-rGO. As GO had a light brownish color and S-rGO a black color suspension, as we have expected, the optical absorption of all S-rGO was higher than that of GO. In agreement with our results, Thakur and Karak observed a characteristics peak value at 268 nm using phytoextracts for both *Camellia sinensis* peel aqueous extract-reduced GO and *Mesua ferrea* leaf aqueous extract-reduced GO [50].

### XRD analysis

**Figure 2:**
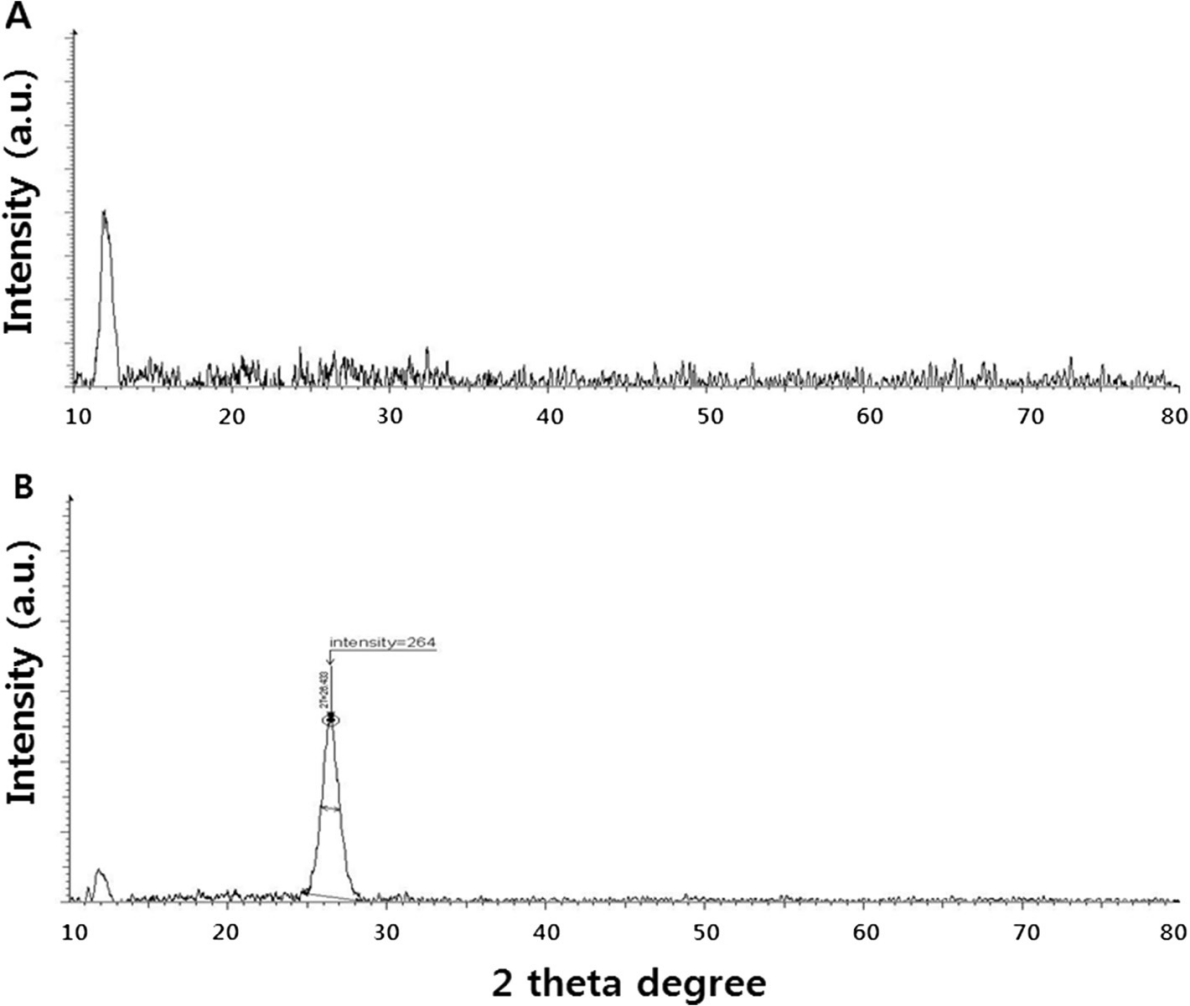
XRD patterns of GO (A) and S-rGO (B).

XRD is an effective technique to investigate the interlayer changes and the crystalline nature of the synthesized material. XRD patterns of GO and S-rGO are shown in Figure 2. Pristine graphite exhibits a basal reflection (002) peak at 2*θ* = 26.6° corresponding to a *d* spacing of 0.335 nm. Upon oxidation of pristine graphite, the (002) reflection peak shifts to a lower angle at 2*θ* = 11.7, (*d* spacing = 0.76 nm). The increase in *d* spacing is due to the intercalation of water molecules and the formation of oxygen-containing functional groups between the layers of the graphite [51]. In contrast with GO, S-rGO shows a broad peak centered at 2*θ* = 26.4° corresponding to a *d* spacing of 0.36 nm which may be due to the restacking of graphene layers. The disappearance of 002 reflection peak of graphite oxide and the appearance of a broad band at 2*θ* = 26.4° in the S-rGOs indicate the formation of few-layer graphene, which are close to that of pristine graphene nanosheets (26.6°), revealing the reduction of graphene oxide by spinach leaf extract. These XRD results suggest that spinach leaf extracts are capable in reducing GO and in removing intercalated water molecules and oxide groups in GO.

### DLS analysis

**Figure 3:**
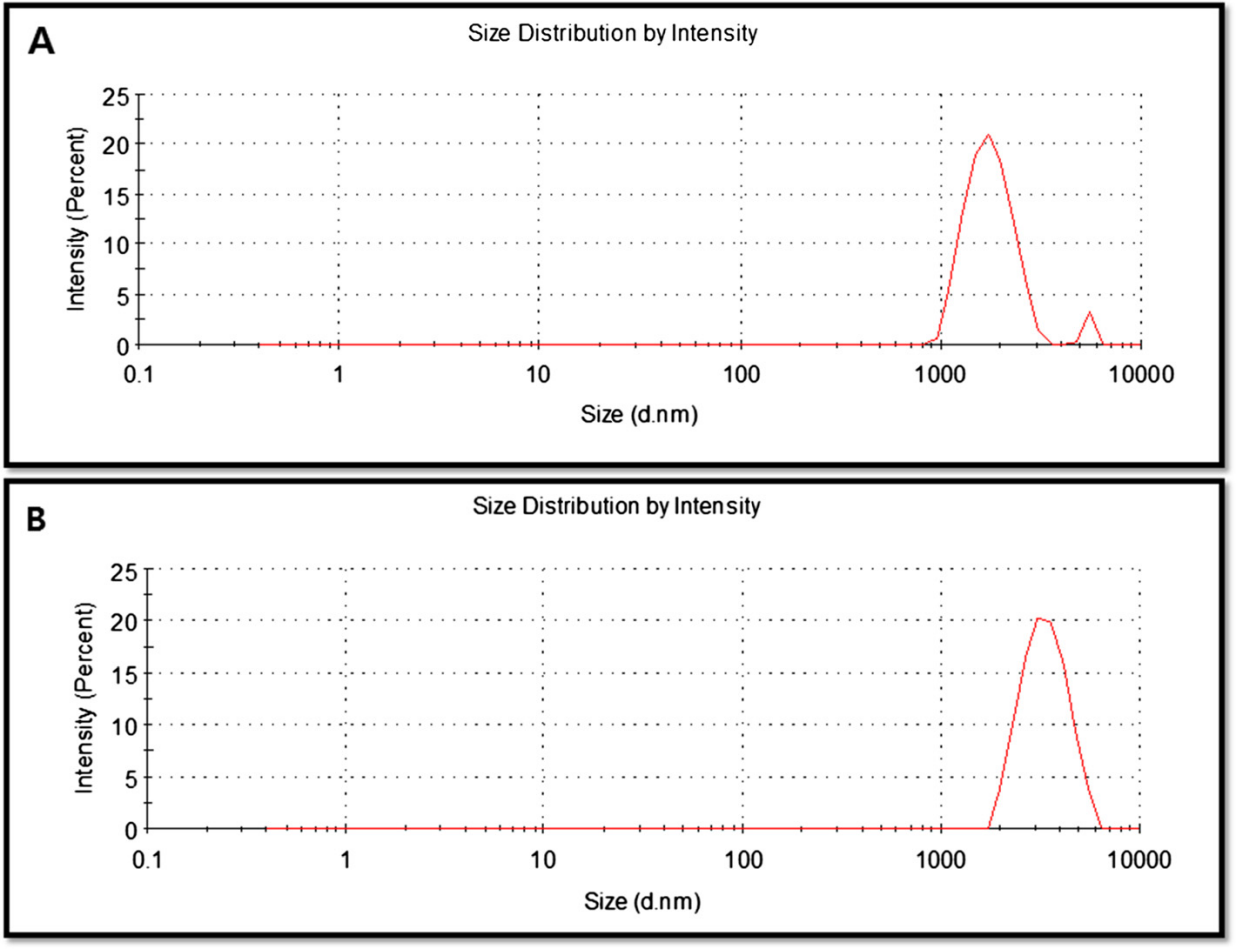
Hydrodynamic size distribution of GO (A) and S-rGO (B).

We employed dynamic light scattering (DLS) technique to elucidate the status of GO and S-rGO sheets in aqueous solution. DLS measurement was performed in aqueous solution to elucidate the size of reduced graphene oxide after reaction with GO. It was found that the average hydrodynamic diameter (AHD) of GO was 2,000 ± 50 nm (Figure 3). However, after the reduction of GO with spinach leaf extract, an AHD of 3,000 ± 70 nm was obtained under the same instrumental conditions, which was relatively higher than that of GO. This noticeable change in size distribution indicated that SLE not only acted as a reducing agent to prepare rGO but also was functionalized on the surfaces of the resulting rGO, leading to an increased size. Stankovich et al. [27] reported that functionalized graphene nanoplates treated with isocyanate show an AHD of 560 ± 60 nm, which is not their average dimension but rather the effective hydrodynamic diameter of an equivalent sphere described by the tumbling of the platelets. Wang et al. [52] reported similar observations using heparin as a reducing agent, and they found that the average sizes of GO and rGO were 302.5 and 392.4 nm, respectively, under the same instrumental conditions, which were relatively larger than that of GO. Liu and coworkers [53] reported that the size of various graphene materials such as Gt, GtO, GO, and rGO dispersions are 5.25, 4.42, 0.56, and 2.93 μm, respectively, and the increasing size could be the aggregation of rGO fragments. The DLS results only show the size differences between GO and rGO [53]. In order to confirm further sizes, the dispersions were further dropped on aluminum foil and dozens of SEM images were taken randomly for each sample.

### FTIR analysis

**Figure 4:**
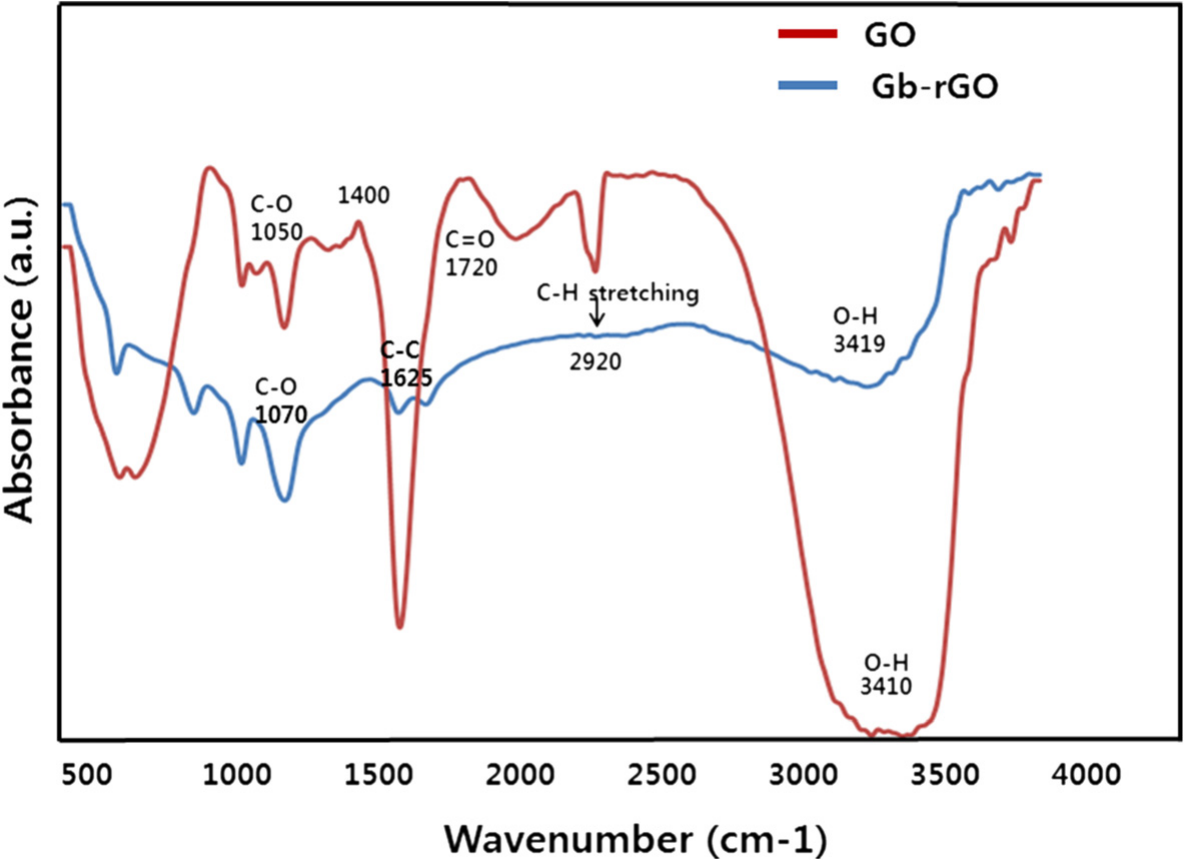
FTIR spectra of GO and S-rGO.

FTIR is a valuable technique to prove the degree of GO reduction. Figure 4 shows FTIR spectra of GO and S-rGO; the characteristic peaks of GO, including O-H stretching at 3,400 cm^−1^, C-O stretching at 1,720 cm^−1^, skeletal vibration of unoxidized graphitic domains at 1,620 cm^−1^, O-H deformation at 1,400 cm^−1^, C-OH stretching at 1,220 cm^−1^, and C-O stretching at 1,030 cm^−1^, were clearly observed in the FTIR spectrum of GO [34,50,51]. As shown in Figure 4, GO has very strong peaks at 3,419 cm^−1^ (O-H) attributed to the water molecules. For the S-rGO sample, the intensities of the bands associated with the oxygen functional groups strongly decreased in relation to those of GO. The results indicate that graphite is successfully oxidized and probably cleaved in the form of GO. GO has two new peaks at 1,720 cm^−1^ (C=O) from carbonyl and carboxylic groups and at 1,050-cm^−1^ (C-O) peak from carbonyl, carboxylic, and epoxy groups, which confirms the presence of oxygen-containing functional groups. The peak at 1,625 cm^−1^ indicates the restoration of sp^2^. The peak at 1,720 cm^−1^ almost disappeared in S-rGO because of the removal of C=O. While being reduced by the extract of leaf, the peaks for oxygen functional groups at 3,400 cm^−1^ significantly decreased. These observations confirmed that most oxygen functionalities in the GO were removed [34,50,51]. The FTIR spectrum of S-rGO indicates a significant reduction of the intensity of all oxygen-containing moieties suggesting an efficient conversion of GO to graphene by the leaf extract of spinach. The obtained results are comparable with earlier report that used various reducing agents for deoxygenation of GO such as sugar [33], tea polyphenol [34,35], and phytoextract [50].

### SEM analysis

**Figure 5:**
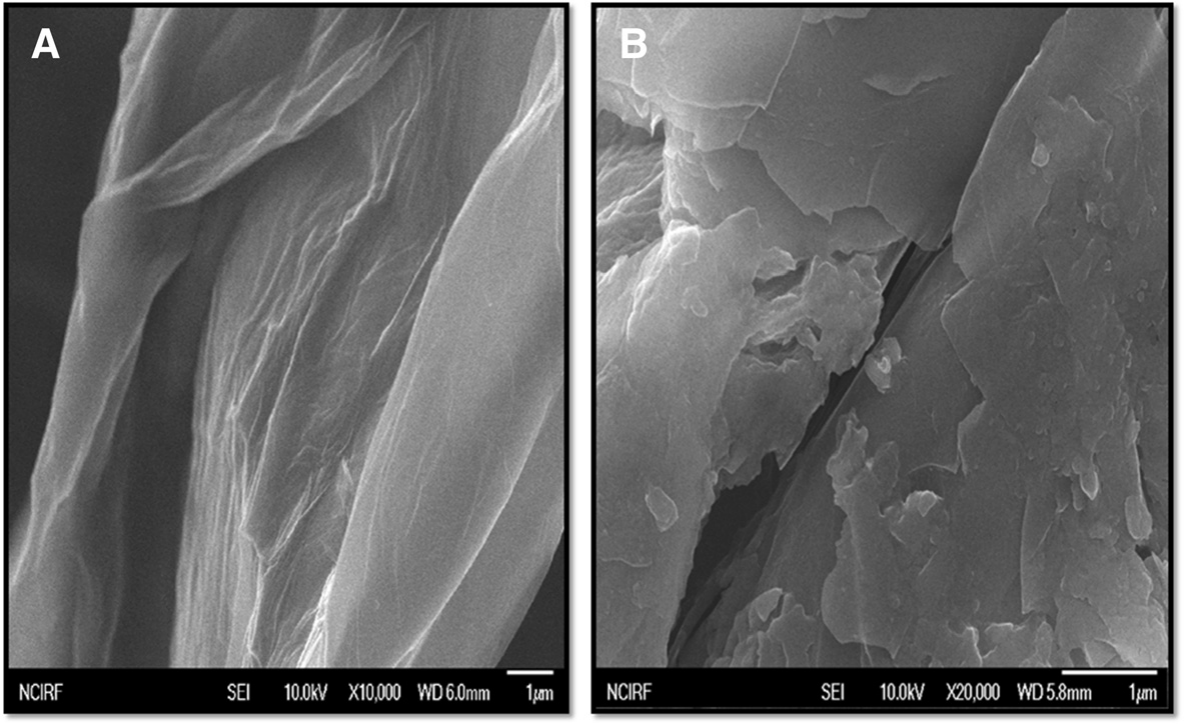
SEM images of GO (A) and S-rGO (B).

The dispersions of GO and S-rGO were further analyzed using SEM. Images were taken randomly from each sample. GO sheets were prepared from natural Gt flakes and had significant solubility in water because of their plentiful oxygen-containing functional groups [54-58]. In general, Gt appears to be piled up with thick cakes, while GO is exfoliated into thin large flakes with wavy wrinkles. The functionalized graphene nanosheets (f-GNs) are mostly wrinkled flakes that are similar to GO, but for the f-GNs functionalized with long chains and polymers, the surfaces are coarse and hairy and the edges of the flakes are blurry [54]. At higher concentrations, the surfaces of GO sheets have a soft-carpet-like morphology, which may be due to residual H_2_O molecules and hydroxyl/carboxyl groups attached to GO [58]. As shown in Figure 5A, GO sheets are smooth with small wrinkles at the edges and also look wavy in nature. The SEM images of GO samples resemble transparent and rippled silk waves. The edges of the exfoliated GO sheets are crumpled due to the oxidation process, whereas S-rGO has a wrinkled paper-like morphology with a sheet-like structure (Figure 5B). As a result of increased levels of oxidation, a significant change was observed at the sharp edges. This difference in morphology between the folded stacked structure of GO and the folded structures for reduced GO implies that the spinach leaf extract reduction process plays a significant role in this transformation of GO to graphene.

### Raman spectroscopy study

**Figure 6:**
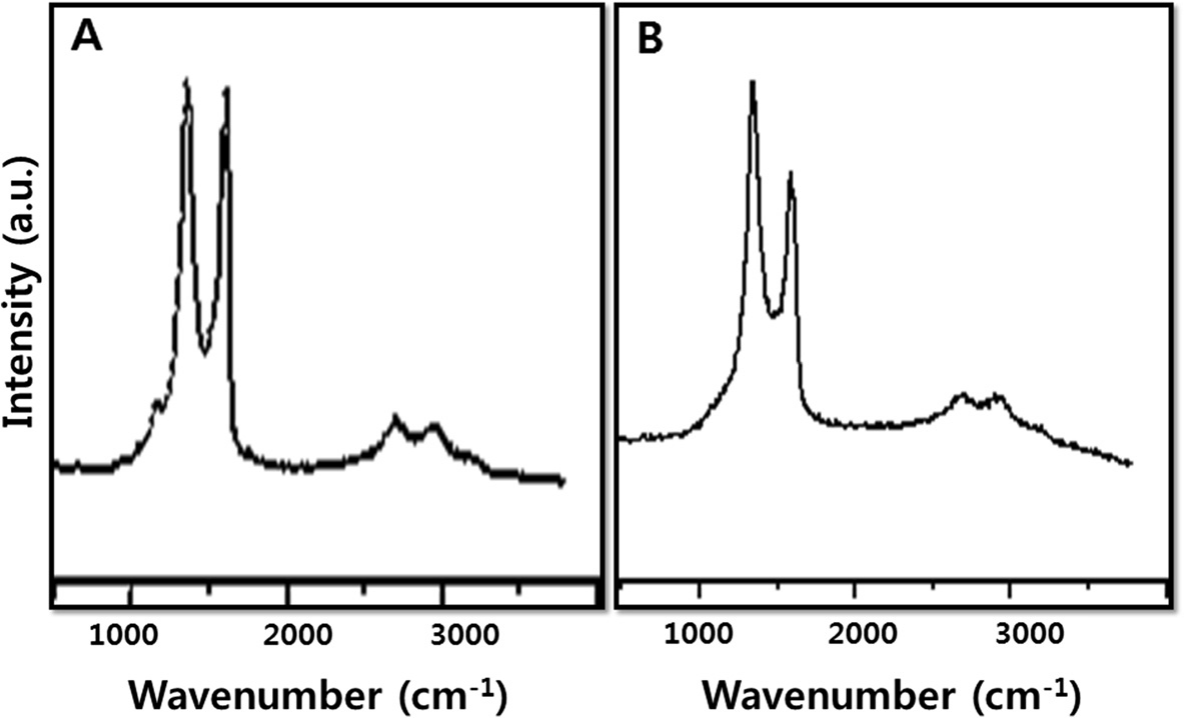
Raman spectra of GO (A) and S-rGO (B).

Raman spectroscopy is an effective tool to characterize graphite and graphene materials, which strongly depend on the electronic structure. As shown in Figure 6A, the Raman spectrum of GO was found to significantly change after the reduction. In the spectra of GO and S-rGO, two fundamental vibration bands were observed in the range of 1,300 to 1,700 cm^−1^. The G vibration mode, owing to the first-order scattering of E_2g_ phonons by sp^2^ carbon of GO and S-rGO, were at 1,611 and 1,603 cm^−1^, respectively, while the D vibration band obtained from a breathing mode of *k*-point photons of A_1g_ symmetry of GO and S-rGO appeared at 1,359 and 1,342 cm^−1^, respectively (Figure 6A,B) [27-29]. After the reduction of GO, the intensity ratio of the D band to the G band (*I*_D_/*I*_G_) was increased significantly, which indicates the introduction of sp^3^ defects after functionalization and incomplete recovery of the structure of graphene [59]. As the D band arises due to sp^2^ carbon cluster, a higher intensity of D band suggested the presence of a more isolated graphene domain in S-rGO compare to GO and that SLE is able to remove oxygen moieties from GO. Wang et al. [60] suggested that the G band is broadened and shifted upward to 1,595 cm^−1^, and increasing the intensity of the D band at 1,350 cm^−1^ could be attributed to the significant decrease of the size of the in-plane sp^2^ domains due to oxidation and ultrasonic exfoliation and partially ordered graphite crystal structure of graphene nanosheets. The Raman spectra of graphene-based materials also show a two-dimensional (2D) band which is sensitive to the stacking of graphene sheets. It is well known that the two-phonon (2D) Raman scattering of graphene-based materials is a valuable band to differentiate the monolayer graphene from multilayer graphene as it is highly perceptive to the stacking of graphene layers [27-29]. Generally, a Lorentzian peak for the 2D band of the monolayer graphene sheets is observed at 2,679 cm^−1^, whereas this peak is broadened and shifted to a higher wave number in the case of multilayer graphene [27-29]. In this investigation, 2D bands were observed at 2,690 and 2,703 cm^−1^ for GO and S-rGO, respectively. The results of the Raman spectrum are in good agreement with those of previous studies in which using aqueous leaf extracts of *Colocasia esculenta* and *M. ferrea* Linn, an aqueous peel extract of orange [50]. Reduced with wild carrot root, the G band of GO is broadened and shifted to 1,593 cm^−1^, while the D band is shifted to a lower region (1,346 cm^−1^) and becomes more prominent, indicating the destruction of the sp^2^ character and the formation of defects in the sheets due to extensive oxidation [51]. This observation is in good agreement with previous studies and supports the formation of functionalized graphene using various biological systems such as baker’s yeast [61], sugar [29,34], and bacterial biomass [38].

### AFM study

**Figure 7:**
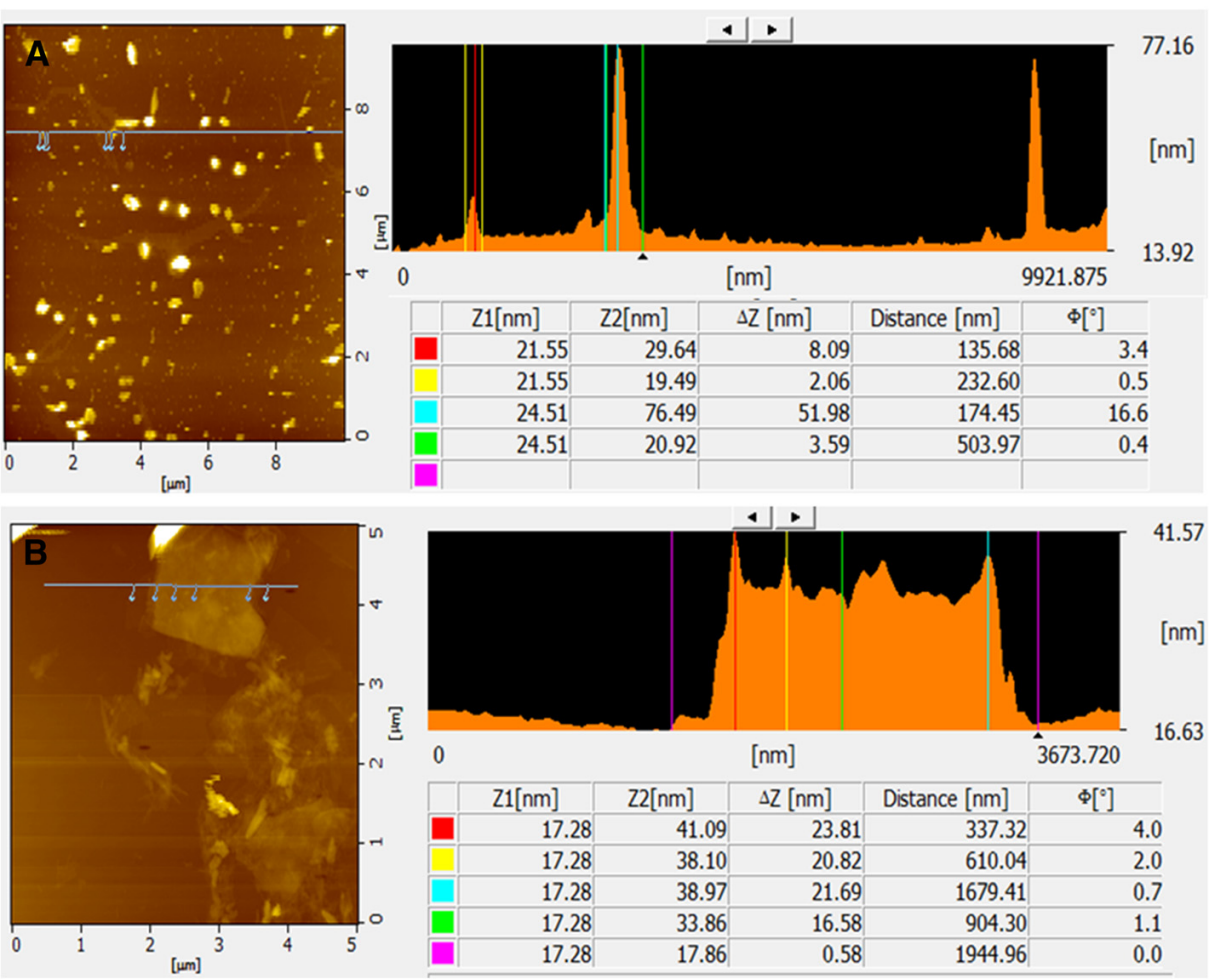
AFM images of GO (A) and S-rGO (B).

Atomic force microscopy (AFM) is an important technique for the morphological characterization of GO and graphene materials and is also capable of imaging and evaluating the surface morphology and properties [54-58]. Figure 7A,B is a typical AFM image of GO and graphene dispersion in water after their deposition on a freshly cleaned glass surface. The average thickness of as-prepared graphene, measured from the height profile of the AFM image, is about 23.81 nm. Compared with the well-exfoliated GO sheets, with a thickness of about 8.09 nm (Figure 7A), the thickness of graphene is larger than that of GO (Figure 7B). The height profile diagram of the AFM image indicates that the thickness of the sheets is around 23.81 nm, comparable to the typical thickness of single-layer GO sheets (8.09 nm). Akhavan et al. [29] used glucose as a reducing agent for the synthesis of graphene and suggested that the increase in thickness of the reduced sheets can be assigned to adsorption of reductant molecules such as glucose-based molecules on both sides of the reduced sheets. Esfandiar et al. [32] observed increased thickness of graphene due to the attachment of the oxidized melatonin molecules on both sides of the reduced GO. Similarly, Zhu et al. [33] suggested that the capping reagent plays an important role in increasing the thickness of the as-prepared GNS, though most of the oxygen-containing functional groups were removed after the reduction. Su et al. [62] demonstrated that dispersed molecules with large aromatic structures and extra negative charges are noncovalently immobilized on the basal plane of graphene sheets via strong interactions.

### Biocompatibility of S-rGO

**Figure 8:**
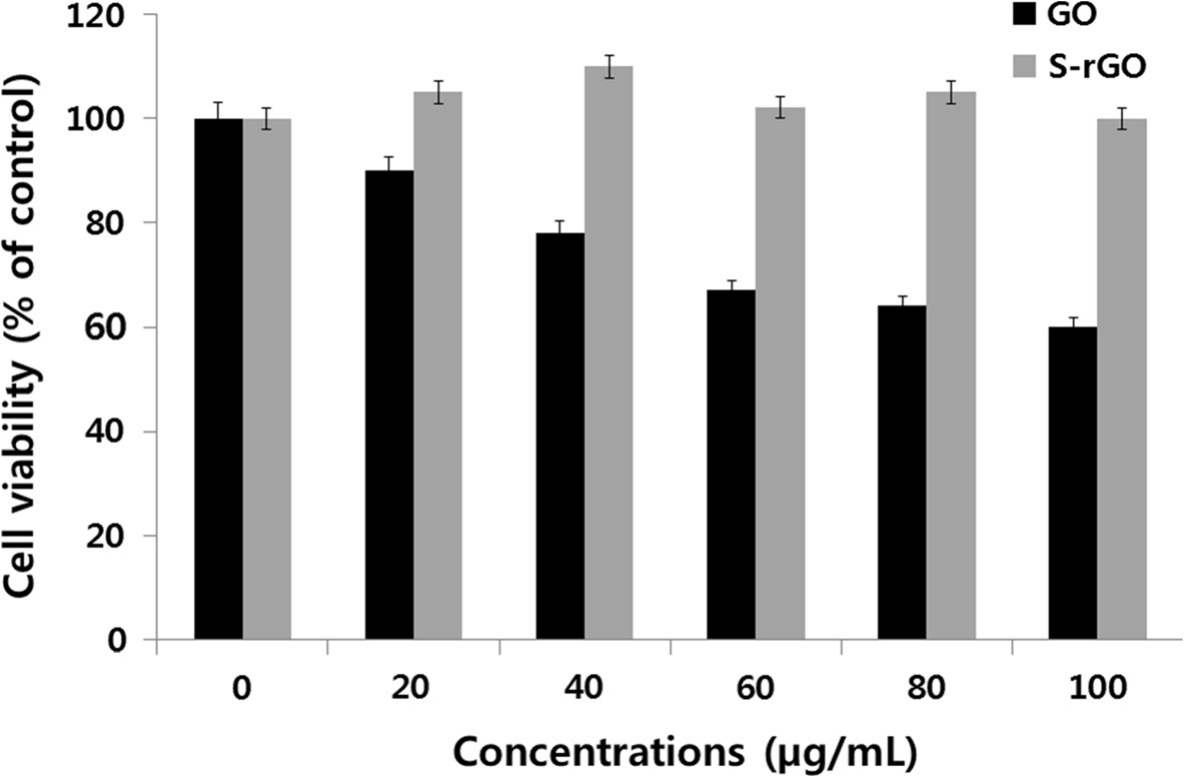
**Effect of GO and S-rGO on cell viability of PMEF cells.** Cell viability of PMEF cells was determined using WST-8 assay after a 24-h exposure to different concentrations of GO or S-rGO. The results represent the means of three separate experiments, and error bars represent the standard error of the mean. GO-treated groups showed statistically significant differences from the control group by Student’s *t* test (*p* < 0.05).

Measuring the biocompatibility of graphene is complex and depends on the techniques used for synthesis and the selection of the biological model system for study. In order to evaluate the biocompatibility of as-prepared S-rGO, the cytotoxic effect of GO and S-rGO against PMEF cells was investigated. As shown in Figure 8, the viability of PMEF cells which were incubated with S-rGO was always around 100% under the used concentrations (10 to 100 μg/mL) after a 24-h exposure. This result indicated that S-rGO was significantly biocompatible even if relatively high concentrations were used; interestingly, cell viability was not compromised when concentrations of S-rGO were increased, whereas when concentrations of GO were increased, the viability decreased to about 40%, which was distinct to S-rGO. Taken together, these results suggested that S-rGO is more compatible than GO which is due to the functionalization of GO by spinach leaf extract. Previous studies demonstrated that hydrazine-rGO was highly toxic to cells [7]. Therefore, it was considered that the surface chemistry was the primary contributor to the difference of toxicity between S-rGO and GO. Several studies have been reported in various cell types such as lung epithelial cells, fibroblasts, and neural cells about the interaction between graphene or GO sheets and cells [11,63-66]. Single-layer GO sheets were internalized in cytoplasmic, membrane-bound vacuoles by human lung epithelial cells or fibroblasts and induced toxicity at doses above 20 μg/mL after 24 h [65]. Recently, Singh and coworkers investigated amine-modified graphene on human platelets, and they found that neither had no stimulatory effect on human platelets nor did it induce pulmonary thromboembolism in mice and suggested that G-NH_2_ is the safest graphene derivative with potential for biomedical applications due to its lack of thrombotic and hemolytic activities. Biocompatibility of graphene films was compared with carbon nanotubes using a mouse fibroblast cell line (L-929) to assess the cytotoxicity; the results suggested that the cells adhered and proliferated on graphene film well than carbon nanotubes, which indicated that the material is biocompatible [67,68]. Akhavan et al. [69] demonstrated that size and concentration are dependent on the cytotoxicity and genotoxicity of graphene oxide sheets and nanoplatelets in the hMSCs and found that the reduced graphene oxide nanoplatelets with average lateral dimensions of 11 nm exhibited a strong potential in the destruction of the cells. The destruction of cells is due to contact interaction of the extremely sharp edges of graphene with the cells, and the possible mechanisms could be oxidative stress which eventually leads to DNA fragmentations and chromosomal aberrations. Furthermore, Akhavan et al. [70] reported that the single-layer reduced graphene oxide nanoribbons could penetrate into the cells and cause DNA fragmentations as well as chromosomal aberrations, even at a low concentration of 1.0 μg/mL after a short exposure time of 1 h in hMSCs.

### Impact of GO and S-rGO on membrane integrity

**Figure 9:**
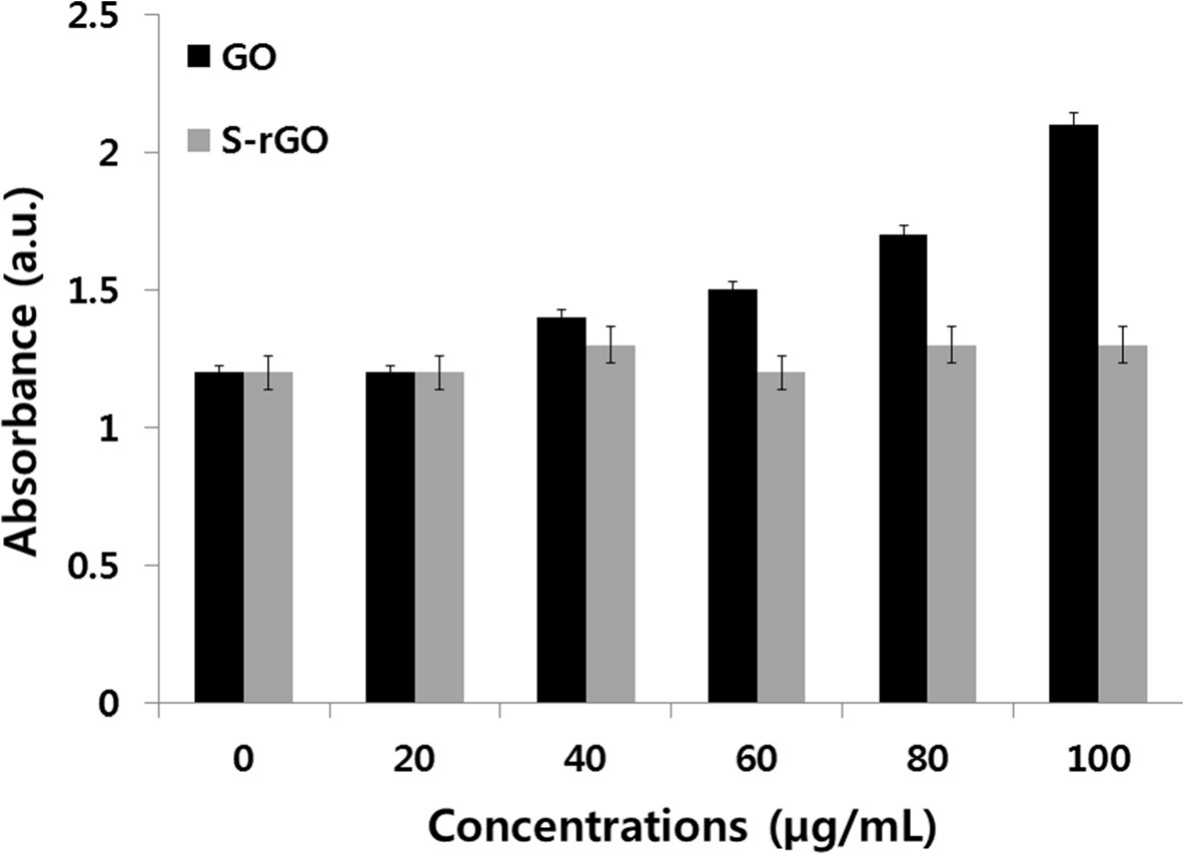
**Effect of GO and S-rGO on LDH leakage in PMEF cells.** LDH leakage was measured by changes in optical densities due to NAD^+^ reduction which were monitored at 490 nm, as described in the ‘Methods’ section, using cytotoxicity detection lactate dehydrogenase kit. The results represent the means of three separate experiments, and error bars represent the standard error of the mean. GO-treated groups showed statistically significant differences from the control group by Student’s *t* test (*p* < 0.05).

The reactive oxygen species (ROS) generated in a concentration-dependent graphene is known as one of the important mechanisms describing the cytotoxicity of graphene [64]. Therefore, because we are interested to evaluate the biocompatibility of GO and S-rGO on cell membrane damage, LDH release (cell membrane damage marker) was measured. As shown in Figure 9, a significant LDH release was observed in the cells treated with GO compared to the control group, and no obvious differences were observed even at higher concentrations of S-rGO treated against the control group. However, distinct increased LDH leakage was observed at higher concentrations between 60 and 100 μg/mL of GO-treated cells. Sasidharan et al. [71] reported that there was no LDH leakage of Vero cells treated with both pristine and functionalized graphene at different concentrations until 300 μg/mL. Recently, Zhang et al. [72] reported that cell cytotoxicity of dispersed nanographene platelets (NGPs) exhibited dose-dependent characters, which had no obvious cytotoxic effects to MG63 cells at a concentration less than 10 μg/mL, whereas it could delay cell cycle, promote cell apoptosis, damage cell microstructure, induce serious tumor necrosis factor-a expression, and greatly reduce ALP activity of MG63 cells at higher concentrations of NGPs. Zhang et al. [63] also reported that a few-layer graphene increased intracellular generation of ROS and induced mitochondrial injury in neural cells after 4 and 24 h at a dose of 10 μg/mL. In contrast, surface-modified graphene and carboxylated graphene were reported to be less toxic than GO or native graphene [73,74].

### Impact of GO and S-rGO on ALP activity

**Figure 10:**
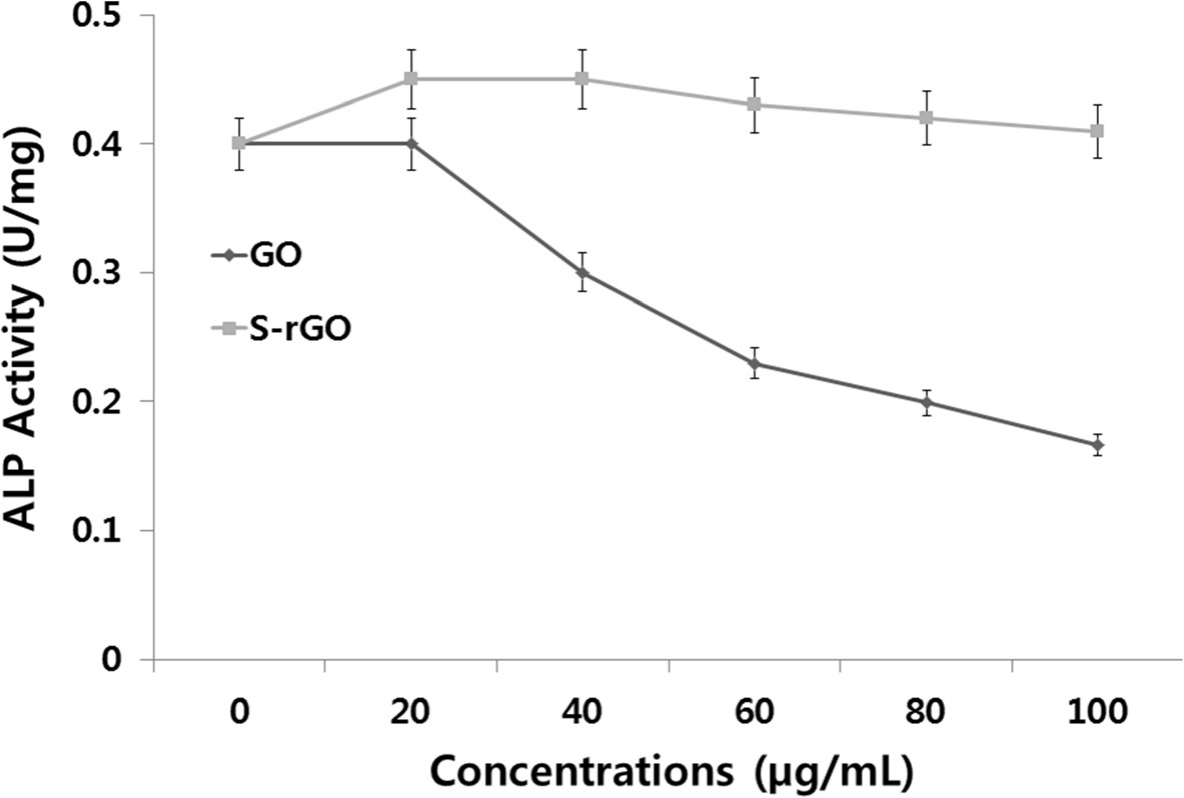
**The effect of GO and S-rGO on alkaline phosphatase activity.** PMEF cells were treated with various concentrations of GO and S-rGO for 4 days. ALP activity was measured as described in the ‘Methods’ section. The results represent the means of three separate experiments, and error bars represent the standard error of the mean. GO- and S-rGO-treated groups showed statistically significant differences from the control group by Student’s *t* test (*p* < 0.05).

ALP activity is an important and quantitative marker of osteogenesis. Furthermore, ALP is an important marker for functional activity of cells such as cell proliferation. Cell numbers and ALP activity were used as measures of cell proliferation, self-renewal, and pluripotency. ALP is a membrane-bound enzyme that exhibits biphasic behavior. It is expressed on the surface of pluripotent undifferentiated ES cells and disappears as cells begin to differentiate. To examine cell differentiation, the ALP was measured as a marker of differentiation. The ALP activity was measured in GO- and S-RGO-treated cells, and the results are represented in Figure 10. Alkaline phosphatase activity was quantified by hydrolysis of p-nitrophenyl phosphate after 4 days of treatment. As expected, GO-treated cells showed a dose-dependent decrease of the alkaline phosphatase activity. The addition of S-rGO significantly enhanced the alkaline phosphatase activity above that of the control or GO-treated groups. Aoki et al. [75] showed significant cell proliferation and ALP activity in single- and multiwall carbon nanotube (CNT)-treated SaoS2 cells, and they suggest that due to the structure and affinity of CNTs toward proteins, CNTs could be the potential scaffold material for tissue engineering. Zhang et al. [72] demonstrated that cells cultured with NGPs at low concentrations have a higher ALP expression close to the negative control group.

## Conclusions

We demonstrated a simple and green approach for the synthesis of water-soluble graphene using spinach leaf extracts. The transition of GO to graphene was confirmed by various analytical techniques such as UV–vis spectroscopy, DLS, FTIR, SEM, and AFM. Raman spectroscopy studies confirmed that the removal of oxygen-containing functional groups from the surface of GO led to the formation of graphene with defects. The obtained results suggest that this approach could provide an easy technique to produce graphene in bulk quantity for generating graphene-based materials. In addition, SLE can be used as an alternative reducing agent compared to the widely used and highly toxic reducing agent called hydrazine. Further, the cells treated with S-rGO show a significant compatibility with PMEF cells in various assays such as cell viability, LDH leakage, and ALP activity. The significance of our findings is due to the harmless and effective reagent, SLE, which could replace hydrazine in the large-scale preparation of graphene. The biocompatible properties of SLE-mediated graphene in PMEFs could be an efficient platform for various biomedical applications such as the delivery of anti-inflammatory and water-insoluble anticancer drugs, and also it can be used for efficient stem cell growth and differentiation purposes.

## Competing interests

The authors declare that they have no competing interests.

## Authors’ contributions

SG participated in the preparation and characterization of GOs and S-rGO. JWH, VE, AAD, DNK participated in culturing, cell viability, LDH assay, and ALP assay. SG and JHK participated in the design and coordination of this study. All authors read and approved the final manuscript.
